# Strategies to implement evidence into practice to improve palliative care: recommendations of a nominal group approach with expert opinion leaders

**DOI:** 10.1186/s12904-015-0044-9

**Published:** 2015-09-29

**Authors:** Jasper van Riet Paap, Kris Vissers, Steve Iliffe, Lukas Radbruch, Marianne J. Hjermstad, Rabih Chattat, Myrra Vernooij-Dassen, Yvonne Engels, on behalf of the IMPACT research team

**Affiliations:** Scientific Institute for Quality of Healthcare (IQ healthcare), Radboud University Medical Center, P.O. Box 9101, 6500 HB Nijmegen, The Netherlands; Department of Anesthesiology, Pain and Palliative Medicine, Radboud University Medical Center, P.O. Box 9101, 6500 HB Nijmegen, The Netherlands; Research Department of Primary Care and Population Health, University College London, Royal Free Campus, Rowland Hill Street, London, NW3 2PF UK; Department of Palliative Medicine, Universitätsklinikum Bonn, Sigmund-Freud-Street 25, 53127 Bonn, Germany; Department of Palliative Care, Malteser Hospital Bonn/Rhein-Sieg, Bonn, Germany; Regional Centre for Excellence in Palliative Care Department of Oncology, Oslo University Hospital, P.O. Box 4956, Nydalen, 0424 Oslo Norway; European Palliative Care Research Centre, Department of Cancer Research and Molecular Medicine, Faculty of Medicine, Norwegian University of Science and Technology, P.O. Box 8905, N-7491 Trondheim, Norway; Department of Psychology, University of Bologna, Viale Berti Pichat 5, 40127 Bologna, Italy; Kalorama Foundation, Nijmegen, The Netherlands

**Keywords:** Palliative care, Europe, Implementation strategies, Quality improvement, Nominal group technique

## Abstract

**Background:**

In the past decades, many new insights and best practices in palliative care, a relatively new field in health care, have been published. However, this knowledge is often not implemented. The aim of this study therefore was to identify strategies to implement improvement activities identified in a research project within daily palliative care practice.

**Methods:**

A nominal group technique was used with members of the IMPACT consortium, being international researchers and clinicians in cancer care, dementia care and palliative care. Participants identified and prioritized implementation strategies. Data was analyzed qualitatively using inductive coding.

**Results:**

Twenty international clinicians and researchers participated in one of two parallel nominal group sessions. The recommended strategies to implement results from a research project were grouped in five common themes: 1. Dissemination of results e.g. by publishing results tailored to relevant audiences, 2. Identification and dissemination of unique selling points, 3. education e.g. by developing e-learning tools and integrating scientific evidence into core curricula, 4. Stimulation of participation of stakeholders, and 5. consideration of consequences e.g. rewarding services for their implementation successes but not services that fail to implement quality improvement activities.

**Discussion:**

The added value of this nominal group study lies in the prioritisation by the experts of strategies to influence the implementation of quality improvement activities in palliative care. Efforts to ensure future use of scientific findings should be built into research projects in order to prevent waste of resources.

## Background

Palliative care is an approach that aims to improve the quality of life of patients facing problems associated with life-limiting illnesses, and their relatives [1]. A growing numbers of new insights and best practices in palliative care are being disseminated via scientific publications and presentations, yet they are often not implemented in daily practice [2]. Failure to implement research findings leads to bias, unnecessary duplication of studies and suboptimal patient outcomes [3, 4]. In the USA, for example, only about 55 % of the patients received recommended care [5].

Chalmers et al. state that about 85 % of the global annual investment in biomedical research is currently wasted, [6] even though effective strategies and models for stepwise implementation of new evidence exist. Examples of such strategies and models include the UK Medical Research Council’s framework for the development and evaluation of complex interventions to improve health, [7] the Plan-Do-Study-Act cycle, [8] or the stepwise implementation model of Grol et al. [2]. Yet the use of such implementation models is often restricted to the time frame after a research project closes. It is a challenge to continue implementation of new evidence and best practices in daily clinical practice after the research or implementation project has been completed, and it is not always seen as the role of researchers [4].

An example of such a project is IMPACT (**IM**plementation of quality indicators in **PA**lliative **C**are s**T**udy). In this EU funded 7^th^ Framework project, quality indicators (QIs) for the organisation of palliative care were developed, and used to assess and improve the organisation of 40 palliative care services across Europe [9]. The results and tools of this project, even though it is built around implementation may not be further disseminated, adopted and implemented as soon as IMPACT finishes, without further action.

The aim of this study was to identify strategies that can facilitate the implementation of scientific output to improve the organisation of palliative care after a large research project like IMPACT has ended.

## Method

A nominal group technique was used. This technique follows a structured and evaluative methodology, developed to facilitate group or team decision making [10]. As such, they can be used to analyse healthcare problems, [11] and bridge the gap between researchers and healthcare professionals [12, 13]. A nominal group differs from focus group interviews as these are often used to explore what individuals believe or feel as well as why they behave in the way they do [14].

### Participants

Participants were members of the IMPACT consortium, all internationally (European) recognised researchers and clinicians (including physicians, nurses, social workers and psychologists) in cancer care, dementia care and palliative care, including (former) board members of the European Association of Palliative Care (EAPC - http://www.eapcnet.eu/) and of Interdem (http://www.interdem.org), an international research group on early detection and timely intervention in dementia. Participants were divided by profession and field of interest to create two groups of similar size and with an equal balance of clinicians and researchers. All participants were aware of the results of the IMPACT project prior to the nominal group sessions, which were: a generic model of palliative care, [13] a set of quality indicators to evaluate the organisation of palliative care, [15] strategies to improve the organisation of palliative care, and an overview of barriers and facilitators of such improvement strategies [16]. At the start of the nominal group sessions, all participants were asked for their consent to participate.

### Conduct of the groups

Both nominal groups were conducted during the annual consortium meeting of the IMPACT project, one day before the EAPC research congress in Lleida (Spain) in June 2014.

Both nominal groups were led by an experienced moderator (YE and MVD, coordinators of the IMPACT project), and an observer was present in each group to take notes. To ensure comparability of the two nominal groups, the moderators discussed the protocol of the nominal group approach prior to commencing the nominal group sessions. As a first step, the moderators invited group members to individually write down strategies for implementing the results of a research project (such as IMPACT) in daily practice, with no limit to the number of strategies. Secondly, the moderators asked each group member to list the strategies in order of importance. These strategies were documented on a flipchart (or laptop and projector) in full view of all participants. Subsequently, the moderators invited the other group members to react to these strategies in order to initiate a discussion. During these discussions, common themes were identified, which allowed the moderators to combine overlapping strategies. When all participants had mentioned their strategies and no further discussion was necessary, the moderators invited each participant to rank their five most important themes (1 being the most important and 5 the least important) and subsequently the feasibility of each theme (1 being most feasible and 5 least feasible). Feasibility was defined as the likelihood that the theme can be operationalised as concrete actions. The ratings provided by the participants were again documented in full view of all participants.

### Analysis

Data resulting from the nominal group technique was analysed using a qualitative approach. In order to merge the recommendations mentioned by participants in the two nominal group sessions, all items mentioned by the participants were independently coded and subsequently compared by two researchers (JRP and YE). They discussed the codes until consensus was reached. When no consensus could be reached, a third researcher was consulted. When all items were coded, categories and themes were derived from the codes. As participants ranked their five most important strategies, this helped in identifying top themes.

### Ethical considerations

The Medical Ethics Committee of the district Arnhem-Nijmegen has declared that this study does not fall within the remit of the Medical Research Involving Human Subjects Act (registration number 2012/075). This means that this study could be carried out without an approval by an accredited medical ethics committee.

## Results

Twenty participants took part in the nominal group (Table 1).

**Table 1:** Participant characteristics

	Group 1	Group 2
Male/Female	6/4	3/7
Age	52.5 (25–64)	56.5 (30–60)
Researcher	5	6
Clinician	5	4
Years in practice (st.dev.)	19 (13.1)	20.7 (12.4)
Type of settings		
Primary care	1	-
Nursing home	-	1
Hospital	4	3
Academic setting	5	6
Country		
Australia	1	-
Belgium	-	1
Finland	-	1
Germany	1	1
Italy	1	1
Netherlands	2	2
Norway	1	3
Spain	1	-
United Kingdom	3	1

In one nominal group, 21 potential strategies were mentioned and in the second nominal group 31. These strategies could be ranked and combined into the following themes (Table 2)

**Table 2:** Strategies mentioned by the participants of both nominal group sessions (themes and categories)

*Dissemination of the results*
• Presentations at conferences
• Publications (e.g. in scientific peer-reviewed journals, in professional journals, via policy channels but also (international) professional organisations, newspapers)
• Social media (e.g. websites, YouTube, Twitter)
*Unique selling points*
• Sales pitch (focus on facilitating factors/preconditions/user friendliness/visibility) • The impact of using unique selling point/strategy/sales pitch
*Educational activities*
• Integration of training activities into daily scheme
• User friendly format (e.g. e-learning modules, mass learning via YouTube, downloadable slides)
• Train the trainer
*Participation of stakeholders*
• Expert organisations (e.g. those responsible for implementation)
• Patients, relatives, professionals and policy makers
• Healthcare insurers and funders •Early adopters: staff in pilot services
*Consideration of consequences*
• Rewards (e.g. financial, certificates)
• Negative consequences (e.g. no accreditation)

### Dissemination of the results

Participants considered conferences, and in particular publications tailored to specific audiences, to be important. For example, results should also be published in ‘policy language’ for policy makers, ‘professional language’ in newsletters of professional scientific organisations and in ‘laymen language’ for the general public (e.g. by using social media).

### Unique selling points

Participants considered a unique selling point or sales pitch a prerequisite to promote quality improvement activities to services. They argued that quality improvement activities should highlight those aspects that are unique, known to be attractive or solve a problem or barrier. Secondly, participants also considered the collaboration between researchers and clinicians an important unique selling point, as this already shows that clinicians have been involved from the start.

### Educational activities

In both nominal groups, participants mentioned using specific e-learning tools as well as mass-learning (e.g. YouTube) for dissemination. They also suggested integration of training activities with daily routines and inclusion of quality improvement activities with the core curricula used by teaching staff.

### Participation of stakeholders

Participants mentioned that it is important to involve different types of stakeholders (patients, professionals, policy makers, insurers and funders). Professionals of a (scientific) organisation can, for example, acknowledge quality improvement activities and include them in their protocols. Therefore, it is important to identify those stakeholders who can and will contribute to quality improvement activities. Participants suggested using local expert organisations, but also ‘early adopters’ derived from the network of the services itself.

### Consequences

Participants mentioned that services can be stimulated to implement quality improvements when they are rewarded for their activities, for example with certificates of best practice. However, they also stated that quality improvement activities require commitment from the service. Services can therefore also be held responsible for failure to implement quality improvement activities. For example, participants suggested threats to the accreditation of services as a sanction for not implementing quality improvement activities.

The strategies mentioned by the participants of both nominal groups led to the following recommendations about implementation strategies to improve the organisation of palliative care (Table 3).

**Table 3:** Recommendations based on the strategies to facilitate implementation

• Publish results regarding the implementation of quality improvement activities tailored to its audience (e.g. patients, professionals caregivers, policy makers and researchers)
• Identify and disseminate unique selling points to implement quality improvement activities
• Develop e-learning tools (e.g. via YouTube)
• Integrate scientific evidence into the core curricula (of practitioner disciplines)
• Stimulate the active participation of important stakeholders to engage and initiate quality improvement activities (e.g. professional (scientific) organisations)
• Reward services that successfully implemented quality improvement activities (e.g. financial incentive)
• Restrict services that provide suboptimal palliative care and do not implement quality improvement activities (e.g. no accreditation)

## Discussion

This study identified specific strategies to implement the results of research projects in the field of palliative care. The nominal group technique allowed international clinicians and researchers to prioritize five common themes: dissemination of the results, unique selling points, educational activities, involvement of stakeholders and the consideration of consequences. These strategies are in line with those found in literature [4, 17–21]. For example, in an overview by Grol and Grimshaw, [18] in which they included 54 reviews about the effectiveness of different interventions to change clinical practice, they described dissemination activities, educational activities and financial interventions. In another overview, Grimshaw et al. described educational activities as well as disincentives [4]. In a review, Giguere et al. described the effect of different strategies using printed educational materials [20]. In a report for the Danish Institute for Health Services Research and Development, Thorsen et al. described dissemination strategies, educational activities and incentives and sanctions [19]. And in his ‘Diffusion of Innovations’, Rogers described the involvement of stakeholders [21]. However, several of the strategies identified have yet to be applied to improve daily clinical practice. For instance, identifying the unique selling points of studies, and using negative consequences for services are methods that are not frequently used. As for negative consequences, in the USA the Centers for Medicare & Medicaid Services applied financial penalties to hospitals that did not improve their hospital acquired infection rate, and as a result infection rates declined in many hospitals [22]. Regarding the participation of stakeholders, it is known that ‘early adopters’ are important: [21] Rogers described that they show a high degree of innovativeness, are a role model for others and help trigger the critical mass when adopting an innovation [21]. But ‘early adopters’ hardly appear to be used when introducing changes into daily clinical practice.

Participants also considered the social media as agents of change that could be used more often. However, various health care organisations, scientific journals, researchers and healthcare professionals use different kind of social media to communicate about palliative care and disseminate new evidence into daily clinical practice [23, 24]. Examples are the EAPC blog (https://eapcnet.wordpress.com/) and reviews of palliative care services (such as available for the Netherlands: https://palliatief.tevreden.nl/). Furthermore, palliative care knowledge networks (such as CareSearch in Australia: http://www.caresearch.com.au/) can contribute to the dissemination and implementation of new evidence in daily clinical practice.

The added value of our study is that all these strategies have been considered together and prioritised by experts.

Surprisingly, de-implementation and mandating quality improvement projects were not mentioned. De-implementation, meaning stopping ineffective or harmful interventions is particularly important as there are many suboptimal forms of care in use. In palliative care, for example, the use of artificial hydration was recently discussed in a paper by Nakajima et al [25]. They showed that artificial hydration did not improve dehydration symptoms, quality of life, or survival in terminally ill cancer patients [25]. The continuation of such ineffective and sometimes even harmful medical practices is undesirable and may result in rising healthcare costs in addition to the increased burden on patients and care givers [26]. Mandating quality improvement projects is important because without the full support of the scientific and professional organisations, the implementation of quality improvement projects will not progress [2].

However, as the participants in our study emphasised, using only one strategy to implement quality improvement activities in daily clinical practice is not sufficient. Participants recommended, in line with Grol and Grimshaw, [18] the use of a combination of strategies. It appears to be important that, for each quality improvement activity a theoretically grounded structured procedure, like the framework for the development and evaluation of complex interventions of the UK Medical Research Council, [7] the Plan-Do-Study-Act cycle, [8] or the stepwise implementation model of Grol et al.,[2] is used. These implementation models facilitate both researchers and professionals in a step-by-step guide to implement evidence based best practices into daily clinical routine. Such models also may provide users with a detailed description of the current situation, preferred situation, reasons why the preferred situation has not been reached, factors that could be used to reach the preferred situation, etc. This information facilitates the development of specific actions necessary to initiate change. For example by following the steps described in the theory of planned behaviour or behaviour change wheel [27, 28]. The recommendations stipulated in this paper can be used to tailor quality improvement activities specifically for palliative care. For example one of the recommendations has already been carried out by organizing the Palliative Care 2020 conference at which stakeholders were invited to discuss the future of palliative care in Europe and which resulted in the European Declaration on Palliative Care [29].

## Conclusion

Research projects generate a growing amount of new knowledge. Often this new knowledge is not implemented in daily practice, particularly in an environment as complex as palliative care. Timely efforts should be made to ensure that the future application of scientific findings is integrated into the research itself, to prevent wasting resources and as an endpoint for better healthcare for patients. The recommendations reported here may be of particular use in promoting quality improvement activities in palliative care. Important stakeholders, such as scientific and professional organizations and leaders on the level where the actual implementation takes place, can perform a key role in the wider implementation of new evidence.

### Strengths and limitations

Strengths of this study were that the IMPACT consortium consisted of an international, multiprofessional group of professionals (including nurses, physicians, social workers and researchers). The mix of researchers with a background in implementation science, professionals active in daily clinical practice and members involved in national policy making, facilitated the identification of optimal implementation strategies. This study allowed the members of the IMPACT consortium to think about future implementation strategies while the study was still ongoing. However, this also shows a limitation of this study; as no patients or informal caregivers were involved.

## Abbreviations

EAPC: European Association of Palliative Care
EU: European Union
IMPACT: IMplementation of quality indicators in PAlliative Care sTudy
Interdem: International research group on early detection and timely intervention in dementia
QI: Quality indicator
UK: United Kingdom
USA: United States of America
